# The Prevalence of Cardiovascular Manifestations in Pediatric Sickle Cell Anemia Patients in a Large Tertiary Care Hospital in the Western Region of Saudi Arabia

**DOI:** 10.7759/cureus.35751

**Published:** 2023-03-04

**Authors:** Rahaf Waggass, Abeer K Alhindi, Inas S Bagabas, Mawaddah H Alsaegh, Noor K Alsharef, Roaa E Morya, Muhammad A Khan, Wasil Jastaniah

**Affiliations:** 1 Pediatric Cardiology, King Saud Bin Abdulaziz University for Health Sciences, Jeddah, SAU; 2 Medicine, King Saud bin Abdulaziz University for Health Sciences, Jeddah, SAU; 3 College of Medicine, King Abdullah International Medical Research Center, Jeddah, SAU; 4 College of Medicine, King Abdullah International Medical Research Center, Jeddah, Saudi Arabia, Jeddah, SAU; 5 Medical Education, King Saud Bin Abdulaziz University for Health Sciences, Jeddah, SAU; 6 Research, King Abdullah International Medical Research Center, Jeddah, SAU; 7 College of Medicine, King Abdulaziz Medical City, Jeddah, SAU; 8 Oncology, King Faisal Specialist Hospital and Research Centre, Jeddah, SAU

**Keywords:** hyperdynamic left ventricle, mitral valve insufficiency, tricuspid valve insufficiency, left ventricular dilatation, pulmonary hypertension, high systolic blood pressure, saudi arabia, children, cardiovascular complications, sickle cell disease

## Abstract

Background

Sickle cell disease (SCD) is a common hematological disorder with a high prevalence in Saudi Arabia. Despite that, limited studies are available in our region regarding cardiovascular complications.

Objectives

The objective of the current study was to estimate the prevalence of cardiovascular complications among children with SCD.

Design

This was a cross-sectional study.

Setting

The study took place at a single tertiary-care center in Jeddah, Saudi Arabia.

Materials and methods

The study reviewed 126 electronic records of pediatric patients up to 16 years old diagnosed with SCD between January 2008 and December 2019 in King Abdulaziz Medical City (KAMC) in Jeddah, Saudi Arabia. Of these, 54 patients had a previous echocardiogram evaluation and were eligible for the study.

Main outcomes measures

The study identified cardiovascular complications in pediatric sickle cell patients.

Sample size

The study included a total of 54 pediatric sickle cell patients.

Results

The mean age was 11.9 (3.48) years, the male-to-female ratio was 2:1, the majority (94.4%) had the HbSS-HbSβ0 genotype, the mean baseline hemoglobin F (fetal hemoglobin) was 20.30 (9.03%), and the clinical severity score was severe in 19 (35.2%) and mild/moderate in 35 (64.8%) patients. Cardiovascular complications occurred in 32 (59.3%) patients. Increased systolic blood pressure was detected in 10 (18.5%) patients. Echocardiography showed left ventricular dilatation in nine (16.7%) patients, tricuspid valve insufficiency in six (11.1%) patients, mitral valve insufficiency in four (7.4%) patients, hyperdynamic left ventricle in one (1.9%) patient, and pulmonary hypertension in one (1.9%) patient. Long QTc interval was noticed in three (5.6%) and cardiomegaly was detected in 18 (33.3%) patients.

Conclusion

Cardiovascular complications occurred at a high frequency in our pediatric population despite high baseline hemoglobin F levels. Early evaluation and continuous monitoring are important for early intervention.

## Introduction

Sickle cell disease (SCD), or sickle cell anemia, is an autosomal recessive disorder characterized by abnormal hemoglobin S (Hb S). This alters the normal round shape of red blood cells (RBCs) [1,2]. It is considered one of the most common genetically passed hematological disorders globally and is associated with high mortality and morbidity. In Saudi Arabia, sickle cell traits range from 2% up to 27% with the highest prevalence in the eastern province followed by the southwestern. Furthermore, up to 2.6% of the population is affected by the disease [2]. In addition, sickle cell disease in Saudi Arabia has two unique haplotypes, the Arab-Indian haplotype in the Eastern region and the Benin haplotype in the Western region. Both haplotypes have a wide variation in the clinical presentation of the disease [2,3].

Sickle cell disease is caused by a mutation at position six of the beta-globin polypeptide chain, resulting in a substitution of glutamic acid with valine, which changes the phenotype of hemoglobin A (Hb A) to Hb S [1,4]. Consequently, the polymerization of deoxygenated Hb S forms aggregates called tactoids, which gives the sickle shape of the RBCs resulting in vaso-occlusion and chronic hemolysis that contribute to increased left-sided volume overload and cardiac output leading to elevated left ventricular filling pressures [1,4,5]. Additionally, sickled RBCs affect oxygen delivery to critical organs, producing episodes of ischemia-reperfusion injury and intravascular hemolysis progressing to chronic hemolytic anemia, cutaneous leg ulceration, renal insufficiency, iron overload, liver dysfunction, and cardiovascular complications [1].

Cardiovascular complications remain one of the major causes of death in sickle cell patients. SCD patients experience a variety of cardiovascular complications, including pulmonary hypertension, ventricular dilatation/enlargement, atrial dilatation, right heart failure, pericardial effusion, cardiomegaly, systolic murmur, and prolonged QTc [1,6-14]. A systematic review was conducted and indicated pulmonary hypertension as one of the commonest manifestations in patients with SCD leading to left ventricular dilation and increased ventricular mass deteriorating into diastolic dysfunction [1].

Vaso-occlusive crisis (VOC), acute chest syndrome (ACS), hepatic sequestration, splenic sequestration, priapism, and cerebral vasculopathy are referred to as sickle cell crises [15]. Cerebral vasculopathy was defined as having a velocity of more than 200 cm/s in transcranial doppler (TCD), a history of silent infarct, or a history of stroke [16].

To our knowledge, limited studies addressing the prevalence of cardiovascular manifestation in pediatric SCD from our region have been conducted. Additionally, two distinct haplotypes of SCD are found in Saudi Arabia, the Arab-Indian and the Benin haplotypes, both have been reported to have a high baseline hemoglobin F level that modifies disease phenotype; therefore, the occurrence of cardiac manifestations may be variable. The objective of this study is to estimate the prevalence of cardiovascular manifestations among a cohort of Saudi children with SCD longitudinally followed at King Abdulaziz Medical City (KAMC), Jeddah.

## Materials and methods

Study design, setting, and participants

This was a cross-sectional study performed in King Abdulaziz Medical City (KAMC), a tertiary care center in Jeddah, Saudi Arabia, and one of the major hospitals in the western region of Saudi Arabia, in which charts of 126 children diagnosed with sickle cell disease between January 2008 until December 2019 were reviewed. The participants were selected from pediatric cardiology and hematology/oncology departments. Participants included in this study were pediatric patients up to 16 years old of both genders diagnosed with SCD who had a previous echocardiogram evaluated by a cardiologist. The sample size was calculated by using the Raosoft® software. Using a non-probability consecutive sampling method to include patients from January 2008 to December 2019, the sample size was estimated at the 95% confidence level with an estimated 30% prevalence of pulmonary hypertension in patients [6] and a margin of error of (0.05). The required minimum sample size was determined to be 91. However, the study was extended to include all 126 patients.

Data collection process

This was a secondary data collection process where the data were collected by the coauthors of this study from the medical records utilizing BestCare software (ezCaretech co., Ltd., Seoul, Korea) and Xcelera software (Philips, Amsterdam, The Netherlands) using a predesigned data collection sheet. Codes were used to ease the process of data entry. The data entry was done by using Microsoft® Excel sheets (Microsoft® Corp., Redmond, WA).

Patients’ demographics obtained were age (years), gender, body mass index (BMI) (kg/m^2^), systolic blood pressure (mmHg), glucose six phosphate dehydrogenase (G6PD), and type of SCD (HbSS, HbSβ0, or others). The collected hematological data were hemoglobin level (gm/dl), mean corpuscular volume (MCV) (fl), and hemoglobin electrophoresis. Hemolytic parameters were lactate dehydrogenase (LDH) (u/l), aspartate aminotransferase (AST) (u/l), total bilirubin (umol/l), and reticulocyte percentage [16]. Sickle cell crises included vaso-occlusive crisis (VOC), acute chest syndrome (ACS), hepatic sequestration, splenic sequestration, priapism, and cerebral vasculopathy [15]. Cerebral vasculopathy was defined as having a velocity of more than 200 cm/s in transcranial Doppler (TCD), a history of silent infarct, or a history of stroke [16]. The severity of sickle cell anemia was assessed using a validated severity index that was adjusted to range from 0-230 instead of a maximum score of 258 points. The items in the index were weighted according to different complications including sickle cell crises, frequency of occurrence, and value of laboratory results [16]. Data on different surgical procedures related to SCD complications were collected such as splenectomy, cholecystectomy, and adenotonsillectomy. Cardiovascular complications, including pulmonary hypertension, dilated left ventricle, valvular insufficiency, hyper-dynamic left ventricle, ventricular dysfunction, long QT syndrome, and cardiomegaly were assessed using echocardiography, electrocardiography, and chest X-ray results, along with routine systolic blood pressure (SBP) measurements to assess an increase in blood pressure, which was defined as blood pressure in the 95th percentile or higher in three or more visits [17]. SBP was measured using a calculator provided by Baylor College of Medicine, Houston, Texas, to automatically adjust for differences in height, age, and gender [18]. In addition, the number of hospitalization and emergency room visits was obtained. Data regarding hydroxyurea, blood transfusion, or iron chelation were documented.

Data analysis

The data were analyzed using the Statistical Package for Social Sciences (SPSS) (SPSS Statistics, Chicago, IL) version 20. For all categorical variables such as gender, SCD genotype and phenotype, the severity of SCD, and cardiovascular complications; frequencies and percentages were reported. For numerical variables, mean and standard deviation were reported.

Ethical consideration

No consent form was needed as this study used chart review for data collection. Participants’ privacy and confidentiality were assured using a coding system and no identifiers were collected. Moreover, all procedures performed in this study were in accordance with the ethical standards of the institutional research committee. The study met all institutional ethical board requirements by King Abdullah International Medical Research Center (KAIMRC) and was approved by the Institutional Review Boards (IRB) number (SP20/045/J).

## Results

Between January 2008 and December 2019, a total of 54 pediatric patients with SCD were included. The clinical characteristics are summarized in Table 1. The mean age at the time of the study was 11.9 (3.48) years and the mean age of SCD diagnosis was 3.49 (2.92) years. The male-to-female ratio was 2:1. The genotype was HbSS-HbSβ0 disease in 51 (94%) of patients. The disease severity index score was mild-moderate in 35 (64.8%) and severe in 19 (35.2%) patients. Hyperactive airway disease was found in 17 (31.5%) patients, and G6PD deficiency was documented in five (9.3%) patients.

**Table 1:** Characteristics of study participants All variables are reported as n (%) unless otherwise stated. BMI: body mass index; SCD: sickle cell disease; G6PD: glucose 6 phosphatase dehydrogenase; Hb: hemoglobin; MCV: mean corpuscular volume; LDL: lactate dehydrogenase; AST: aspartate transaminase

Demographic Variables	n=54
Age, mean (SD) (years)	11.9 (3.48)
BMI, mean (SD)	17.54 (3.62)
Gender	
Female	18 (33.3)
Male	36 (66.7)
Age at diagnosis of SCD, mean (SD) (years) (n=51)	3.49 (2.92)
Genotype	
HbSS-HbSB0	51 (94.4)
Others	3 (5.6)
SCD severity	
Mild-moderate	35 (64.83)
Severe	19 (35.2)
Comorbidities	
Hyperactive airway disease	
No	37 (68.5)
Yes	17 (31.5)
G6PD deficiency	
No	49 (90.7)
Yes	5 (9.3)
Baseline Hematological Parameters	
Hb level, mean (SD) (g/dl)	8.70 (1.56)
HbS %, mean (SD)	74.15 (8.87)
HbF %, mean (SD)	20.30 (9.03)
HbA %, mean (SD)	1.17 (4.97)
HbA2 %, mean (SD)	3.11 (1.04)
MCV, mean (SD) (fl)	83.97 (11.97)
Hemolytic Parameters	
LDL, mean (SD) (u/l) (n=51)	449.75 (197.40)
Reticulocyte percentage, mean (SD)	10.79 (7.51)
Bilirubin, mean (SD) (umol/l) (n=52)	41.34 (46.51)
AST, mean (SD) (u/l) (n=52)	39.38 (12.78)

Hematological parameters revealed a mean Hb of 8.70 (1.56) g/dl, mean HbS of 74.15 (8.87), and mean HbF of 20.30 (9.03%). Hemolytic parameters showed a mean lactate dehydrogenase (LDH) of 449.75 (197.40) u/l, mean reticulocyte percentage of 10.79 (7.51%), mean bilirubin 41.34 (46.51) umol/l, and mean aspartate aminotransferase (AST) of 39.38 (12.78) u/l.

The clinical course and non-cardiovascular complications of patients included in this study are summarized in Table 2. The history of emergency room visits was documented in 50 (92.6%) patients while the history of admissions was found in 49 (90.7%) patients. The therapeutic intervention showed that hydroxyurea was used by 48 (88.9%) patients with a mean starting age of 7.49 (3.30) years. A history of blood transfusion was noticed in 43 (79.6%) patients with intermittent transfusion being the most common mode in 34 (81%) patients. Iron chelation was administered to five (9.3%) patients.

**Table 2:** Additional characteristics of study participants All variables are reported as n (%) unless otherwise stated. HU: hydroxyurea; VOC: vaso-occlusive crisis; ACS: acute chest syndrome

Demographic Variables	n=54
ER visits	
No	4 (7.4)
Yes	50 (92.6)
Admission	
No	5 (9.3)
Yes	49 (90.7)
Therapeutic intervention	
Hydroxyurea	
Used at least once	
No	6 (11.1)
Yes	48 (88.9)
Age at starting HU (years), mean (SD) (n=47)	7.49 (3.30)
Transfusion	
Received blood transfusion	
No	11 (20.4)
Yes	43 (79.6)
Type of transfusion (n=42)	
Intermittent Transfusion	34 (81.0)
Chronic Transfusion	4 (9.5)
Temporizing	4 (9.5)
Iron Chelation	
No	49 (90.7)
Yes	5 (9.3)
Bone Marrow Transplantation	
No	52 (96.3)
Yes	2 (3.7)
SCD crises	
Ever had SCD crises	
No	5 (9.3)
Yes	49 (90.7)
VOC (≥ 3 episodes)	
No	10 (18.5)
Yes	44 (81.5)
ACS (> 2 episodes)	
No	42 (77.8)
Yes	12 (22.8)
Splenic sequestration	
No	41 (75.9)
Yes	13 (24.1)
Pain crisis	
No	33 (61.1)
Yes	21 (38.9)
Aplastic crisis	
No	47 (87.0)
Yes	7 (13.0)
Hemolytic crisis	
No	42 (77.8)
Yes	12 (22.2)
Cerebral vasculopathy	
No	49 (90.7)
Yes	12 (22.8)

An SCD crisis that required admission was recorded in 49 (90.7%) patients. These crises include VOC, ACS, splenic sequestration, hepatic sequestration, pain crisis, and cerebral vasculopathy. A total number of three or more episodes of VOC were reported in 44 (81.5%) patients. A total number of more than two episodes of ACS was documented in 12 (22.8%) patients. Splenic sequestration was found in 13 (24.1%), pain crisis in 21 (38.9%), and cerebral vasculopathy in five (9.3%) patients.

Cardiovascular abnormalities, as shown in Table 3, were detected in 32 (59.3%) patients. Increased systolic blood pressure of more than the 95th percentile was recorded in 10 (18.5%) patients. Echocardiography showed; left ventricular dilatation in nine (16.7%) patients, tricuspid valve insufficiency in six (11.1%) patients, mitral valve insufficiency in four (7.4%) patients, hyperdynamic left ventricle in one (1.9%) patient, and pulmonary hypertension in one (1.9%) patient. A long QTc interval was noticed in three (5.6%) patients. Cardiomegaly was detected in 18 (33.3%) patients.

**Table 3:** Cardiovascular complications

Cardiovascular complications	All patients (N=54) n (%)
Presence of cardiovascular complication	
No	22 (40.70)
Yes	32 (59.3)
Pulmonary hypertension	
No	53 (98.1)
Yes	1 (1.9)
Dilated left ventricle	
No	45 (83.3)
Yes	9 (16.7)
Tricuspid valve insufficiency	
No	48 (88.9)
Yes	6 (11.1)
Mitral valve insufficiency	
No	50 (92.6)
Yes	4 (7.4)
Hyperdynamic left ventricle	
No	53 (98.1)
Yes	1 (1.9)
Ventricular dysfunction	
No	54 (100)
Yes	0 (0)
Long QTc interval	
No	8 (14.8)
Yes	3 (5.6)
Not done	43 (79.6)
Cardiomegaly	
No	35 (64.8)
Yes	18 (33.3)
Not done	1 (1.9)
Increased systolic blood pressure >95^th^ percentile	
No	44 (81.5)
Yes	10 (18.5)

**Figure 1:**
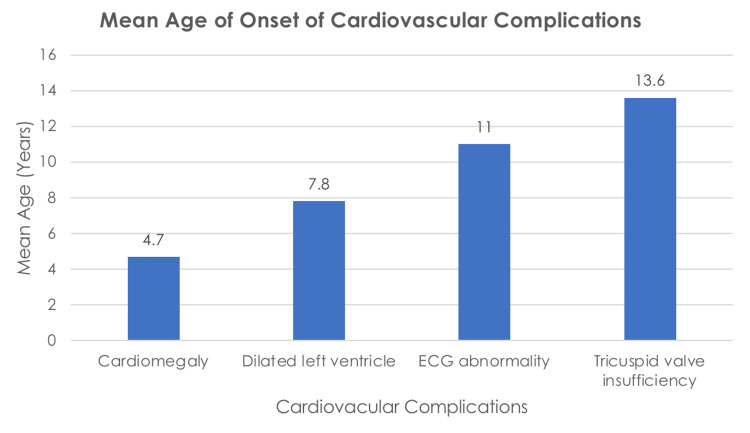
Mean age of onset (in years) of cardiovascular complications

As observed in Table 4 and Figure 1, the mean age of patients with cardiomegaly was 4.7 (3.16) years. The mean age of children with normal echocardiography was 7.6 (3.94) years, and the mean age of children with abnormal echocardiography was 8.5 (4.90) years. Left ventricle dilatation was detected at a mean age of 6.8 (2.68) years, and the mean age of tricuspid valvular insufficiency was 13.6 (2.97) years. The mean age of children with a normal ECG was 4.1 (1.96) years while the mean age of long QTc was 11.0 (5.23) years.

**Table 4:** Mean age in years of normal and abnormal results

	n	Mean (SD)
Age of normal echocardiography	39	7.6 (3.94)
Age of abnormal echocardiography	14	8.5 (4.90)
Age of dilated left ventricle	9	7.8 (2.68)
Age of tricuspid valve insufficiency	5	13.6 (2.97)
Age of normal ECG	8	4.1 (1.96)
Age of abnormal ECG	4	11.0 (5.23)
Age of normal X-ray	35	9.9 (4.51)
Age of abnormal X-ray (cardiomegaly)	18	4.7 (3.16)

## Discussion

Sickle cell disease is one of the major health conditions in Saudi Arabia that has resulted in variable systemic complications, including the cardiovascular system. This study aimed to measure the prevalence of cardiovascular complications in a cohort of Saudi children with SCD as limited studies have addressed this complication previously.

Cardiovascular complications were detected in 59.3% of the studied population. This high percentage can be explained by the fact that cumulative cardiovascular complications were reported, in contrast to other studies that reported each complication separately. In the current study, the most frequently reported complication was cardiomegaly, followed by increased systolic blood pressure, dilated left ventricle, tricuspid valvular insufficiency, mitral valve insufficiency, long QTc interval, pulmonary hypertension, hyperdynamic left ventricle accounting for the same percentage.

Cardiomegaly was observed in 33.3% of the studied population, which was similar to a study in Oman reporting cardiomegaly in 39% [9]. This can be reasoned by the increased volume overload that is caused by chronic anemia resulting in chamber enlargement and increased wall thickness [9]. In addition, we found that increased systolic blood pressure was observed in 18.5% unlike a study conducted in Qatif, Saudi Arabia, which revealed that there was no evidence of systemic hypertension in 87 patients at a mean age of 7.8 years [19]. This could be attributed to the demographic variability reported in the population studied compared to the current study. The mean baseline hemoglobin was 9.6 g/dl vs. 8.7 g/dl, the mean Hb F% was 23% vs 20.3%, and the incidence of G6PD was 32% vs 9.3% in the study from Qatif vs. the present, respectively.

A dilated left ventricle is one of the most common causes of increased morbidity and mortality in sickle cell pediatric patients as documented in a systematic review [1]. In our study, a dilated left ventricle was found in 16.7%; however, studies done in Qatif, Saudi Arabia, and Philadelphia, United States showed 21% and 25%, respectively [19,20]. In Philadelphia, the study included 172 patients with a mean age of 15 years, with the majority (72%) having severe phenotype, as opposed to our study in which the majority (65%) had mild-moderate phenotype. Additionally, the percentage of dilated left ventricles in Michigan, United States was 42.4% in 73 patients at a mean age of 10.1 years and a mean Hgb F of 10.1% [21]. Furthermore, dilated LV in Sudan was reported in 51% of 289 patients with the majority 52% having severe disease [10].

A long QTc interval was noticed in 5.6% in comparison to a study conducted in Chicago that showed a percentage of 38% [13]. The hyperdynamic left ventricle was observed in 1.9%. Tricuspid valvular insufficiency was observed in 11.1% while mitral valve insufficiency showed a percentage of 7.4%. In the United States, a study found that the mean tricuspid regurgitation velocity (TRV) was 2.3 m/s in 399 patients with a mean age of 12 years and the study found that higher regurgitation velocity was associated with high Hgb F, which causes hypoxia and increased erythropoietin concentration. Therefore, decrease tissue oxygen delivery due to high Hgb F and increased erythropoietin were found to be associated independently with pulmonary hypertension (defined as TRV ≥ 2.5 m/s) [22].

Pulmonary hypertension was detected in 1.9% unlike other studies conducted in Qatif, Saudi Arabia, and Philadelphia, United States, which showed no evidence of pulmonary hypertension [19,20]. However, a study done in the United States showed that 57.7% had pulmonary hypertension of which 52 patients were enrolled with a mean age of 16.21 years [23]. This was attributed to the presence of a previous history of pulmonary disease, which included acute chest syndrome, obstructive sleep apnea, reactive airway disease, or asthma in 75% of the patient [23].

In the present study, the mean age of the identification of the different cardiac complications ranged from 4.7 years to 13.6 years, with a standard deviation ranging from three to five years. Cardiomegaly (Figure 1) was the earliest reported problem at a mean age of 4.7 years, followed by dilated left ventricle at a mean age of 7.8 years, ECG abnormality at a mean age of 11 years, and tricuspid valve insufficiency at a mean age of 13.6 years. This suggests that early screening starting in the first five years of life with five-year screening intervals for asymptomatic patients may be justified for early detection and intervention.

This study was limited by the fact that cardiovascular complications could not be reported in more than 50% of the studied population in whom echocardiography was not performed. Despite limited participants, the study results are nonetheless valid for the purpose of answering our research question. In our study, the mean baseline HbF was found to be high. This could be explained by the fact that in our region, the Arab-Indian haplotype of SCD is prevalent. Despite that, there was a high frequency of cardiovascular complications observed in the studied population which ultimately highlights the importance of early screening for cardiovascular complications.

## Conclusions

The finding of this study showed a high frequency of cardiovascular complications in our pediatric population despite high baseline hemoglobin F levels. The most frequent cardiovascular complication was cardiomegaly followed by increased systolic blood pressure and a dilated left ventricle. Early detection and continuous monitoring of such complications are needed to prevent the latent risk of morbidity and early mortality associated with cardiac complications.
